# Gapless edge states in (C,O,H)-built molecular system with p-stacking and hydrogen bonds

**DOI:** 10.1038/s41598-017-09954-z

**Published:** 2017-08-29

**Authors:** Małgorzata Wierzbowska

**Affiliations:** Institute of High Pressure Physics, Polish Academy of Sciences, Sokołowska 29/37, 01-142 Warsaw, Poland

## Abstract

The gapless edge states have been found in a 2D molecular system built with light atoms: C,O,H. This prediction is done on the basis of combined density functional theory (DFT) and tight-binding calculations. The system does not exhibit any effect of the spin-orbit coupling (SOC), neither intrinsic nor Rashba type. The band structure and the edge states are tuned with a strength of the p-stacking and O...H interactions. The elementary cell of this noncovalent structure, does not have the 3D inversion or rotational symmetry. Instead, the system transforms via a superposition of two reflections: with respect to the *xz* and *xy* mirror planes, both containing the non-periodic direction. This superposition is equivalent to the inversion in the 2D subspace, in which the system is periodic. The energy gap obtained with the DFT method is 0.11 eV, and largely opens (above 1 eV) with the GW and hybrid-DFT approaches. The bands inversion is partial, i.e. the Bloch states are mixed, with the ”swapping” and ”non-swapping” atomic contributions.

## Introduction

Topological insulators (TIs) attracted much attention, starting from the work by Kane and Mele^1^ in 2005 to the Nobel Prize for David J. Thouless, F. Duncan M. Haldane and J. Micheal Kosterlitz in 2016. This is due to many plausible applications of their metallic surface states, with the linear dispersions, propagating electrons without the elastic scattering^2^. First experimentally found TIs were HgTe/CdTe^3^ and InAs/GaSb^4^ quantum wells, followed by the 3D crystals with the time-reversal symmetry, such as Bi_2_Sb_3_, Bi_2_Te_3_ and Sb_2_Te_3_^5, 6^. Further, the Heusler compounds containing rare-earth elements^7^, (Tl, Hg, Pb, Cu, Ag)-chalcogenides^8, 9^, Zintl compounds^10^, antiperovskites^11^ and strongly correlated oxides^12^ have been found. All contained heavy elements, and some of them were under pressure^9^. The spin-orbit coupling in these materials is a key-parameter, because it is responsible for the bands inversion and opening of the gap^13^. The 2D TIs, such as a single and multilayer Bi^14, 15^, silicene on the silver surface^16^, electrically controlled multilayer germanene^17^, oxygen-functionalized MXenes^18, 19^, arsenene monolayers^20^, SiTe^21^ and porous MoS_2_^22^ were discovered. Then, a few organic networks were theoretically predicted to possess the topological edge states. Their list contains: triphenyl-lead^23^, s-triazines^24^, nickel-bis-dithiolene^25^ honeycomb lattices, and ligand complexes of Pd and Pt^26^.

In 2011, a new class of TIs was theoretically postulated, namely topological crystalline insulators (TCIs)^27^. They were supposed to be built with light atoms and not show any effect of SOC. These materials have the doubly degenerate (due to spin) surface states with quadratic dispersions. They are protected by time-reversal and discrete rotational symmetry^27–29^ (e.g. *C*_6_ and *C*_4_ in 3D). The electron’s orbital degrees of freedom should play there a role similar to spin. In 2014, the group of TCIs was theoretically extended to the 3D systems without the time-reversal symmetry, but possessing the rotational symmetries with the mirror planes (i.e. *C*_*nv*_ for n = 3, 4, 6, and not 2)^30^.

Early reports about TCIs, based on numerical simulations, predicted the gapless edge states in PbSe, PbTe, PbS^31^ and pyrochlore oxides A_2_Ir_2_O_7_ (A = rare-earth element)^32^. The strong ionic character and SOC were responsible for the bands inversion in these materials. Later, SnSe and SnS were theoretically proposed as TCIs^33^. Although the SOC was still present, it did not play a main role in the topological mechanism of the bands inversion, but it just opened the gap. The experimental reports confirmed the TCI phase in SnTe^34, 35^ and Pb_1−*x*_Sn_*x*_Se/Te alloys^36–38^. The 2D TCIs have been predicted in TlS and TlSe^39^, SnTe^40^, PbSe^41^, containing heavy atoms. The 3D heterostructure of 2D materials PbSe/h-BN was postulated, on the base of the mirror symmetry consideretions, to possess the TCI phase^42^. It was theoretically suggested, that the topological order and Rashba SOC can be tuned with the hydrostatic pressure, epitaxial strain, and additionally controlled with the electric field, in the antiferroelectric *ABC*-compounds^43^. In contrast, up to date, there is probably only one theoretical report on the 2D TCI system with a negligible SOC, namely the twisted graphene multilayers^44^.

**Figure 1:**
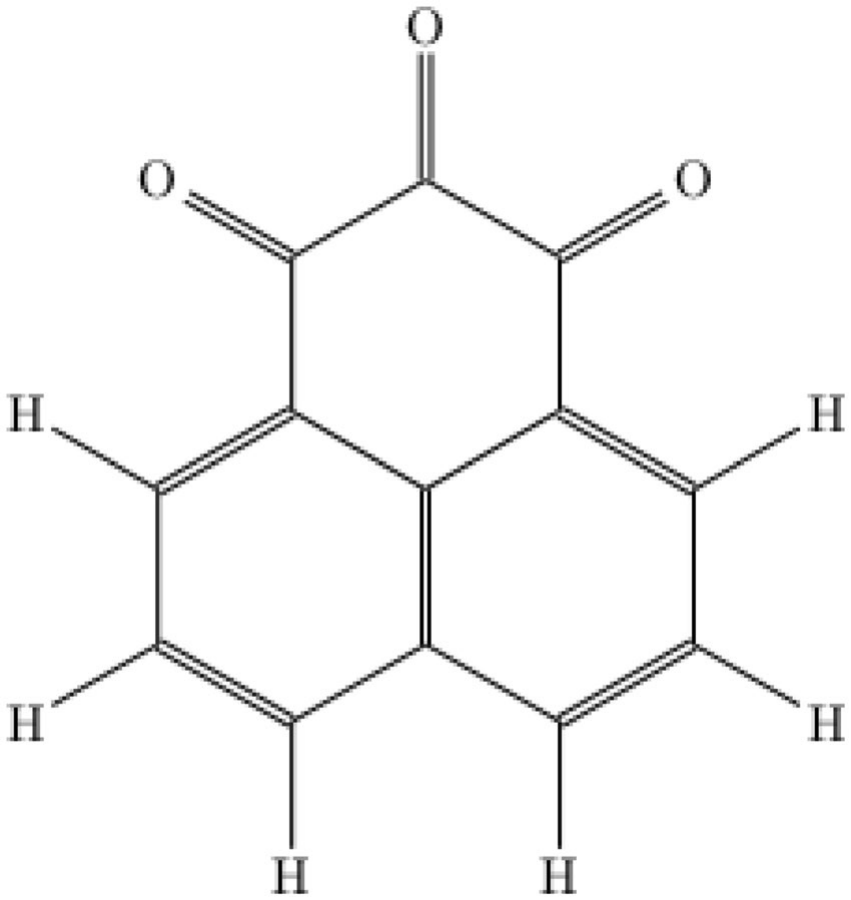
The chemical structure of the studied phenalene derivative.

In this work, the gapless edge states were found in a 2D molecular crystal composed of phenalene derivative. The chemical structure of this molecule is presented in Fig. 1. It is not aromatic, because a complete termination of one C-ring with oxygens fixes a pattern of the double bonds. Similarity of the studied molecule to 1H-phenalene-1,3(2H)-dione^45^ makes an expectation that it could be synthesized.

In a plausible TCI reported here, the molecules are *π*-stacked with a given order. Their columns are periodically repeated in such a way, that the hydrogen bonds form between them along one of the planar directions. The edge states are tuned with two intermolecular distances: between the molecular planes (*d*_*π*-*stack*_) and between the O= and H-terminations (*d*_*OH*_) of the neighbouring molecules.

## Results

Topological band effects show up when non-aromatic molecules are *π*-stacked in a given geometrical order. By applying a pressure along the molecular columns, one can pass from the insulating to metallic state. In some cases the gapless edge states, between two “normal” phases, appear.

**Figure 2:**
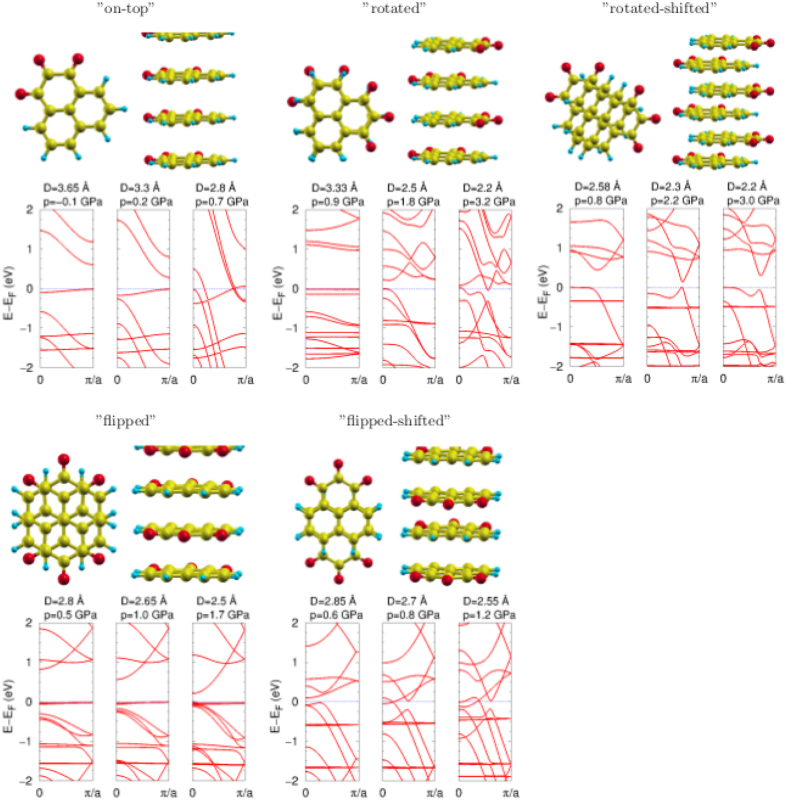
The geometries of five molecular wires, called accordingly to the stacking patterns: “on-top”, “rotated”, “rotated-shifted”, “flipped” and “flipped-shifted”, as well as their band structures, for three intermolecular stacking distances. The pressures are given above the corresponding panels. The Fermi level is at zero energy.

Figure 2 shows five orders of the 1D stacking: “on-top”, “rotated”, “rotated-shifted”, “flipped” and “flipped-shifted”. The corresponding band structures of the wires, at three chosen intermolecular distances, are also displayed. It is interesting to note, that for two orders, namely “on-top” and “rotated”, the metallic phase forms under pressure. While for the third one, called the “rotated-shifted” structure, two different “normal” insulating phases are separated by the “topological” phase. These states are followed by a metal phase with the negative gap, at a very high pressure. The band structure of the “flipped” order has a semiconducting character. While for the “flipped-shifted” order, the semiconducting phase transforms into the metallic phase at quite low pressure. Thus, further in this work, only the “rotated-shifted” order is examined, as a potential topological insulator.

**Figure 3:**
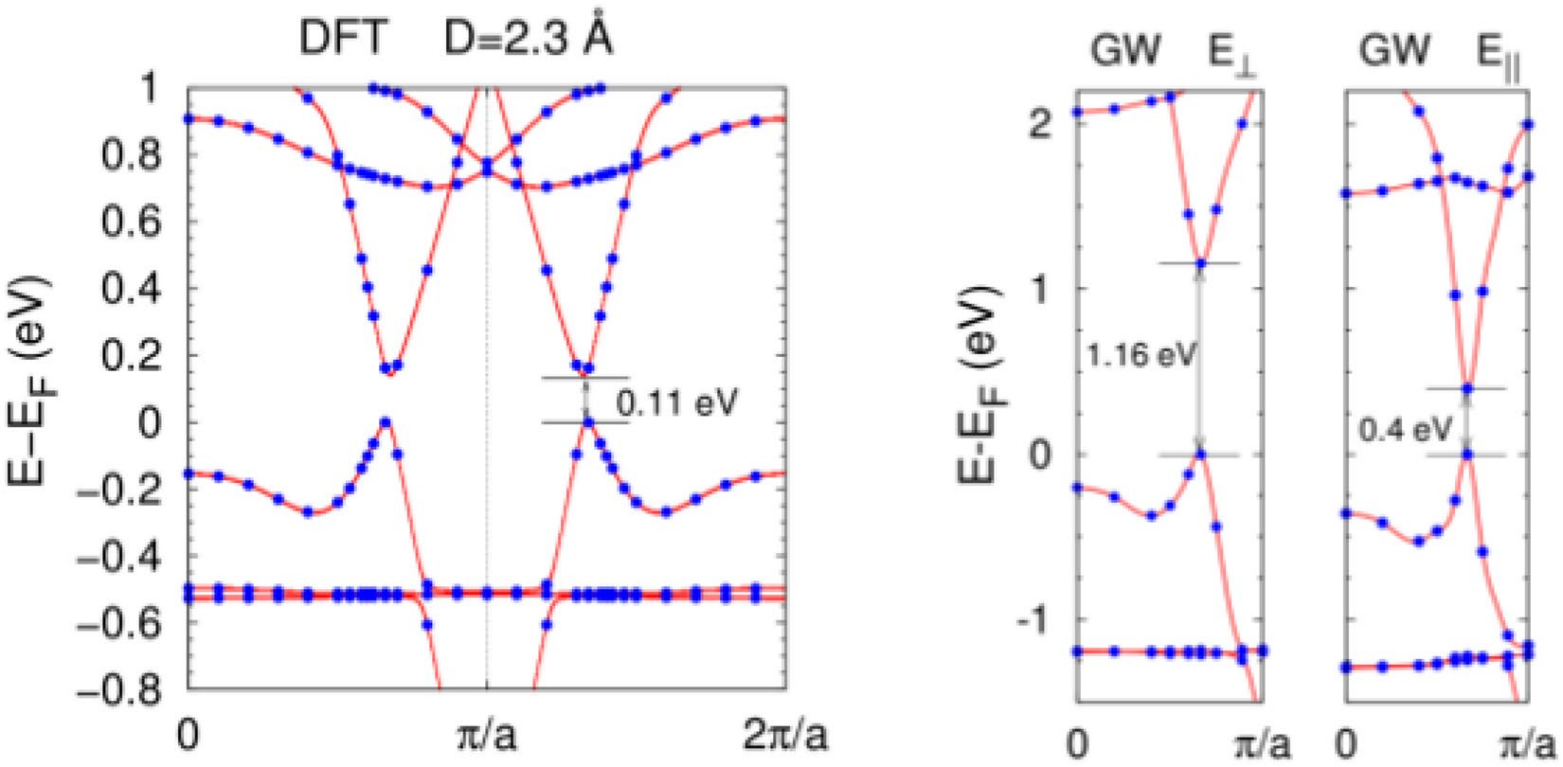
Band structures of a wire with the “rotated-shifted” molecular order, for a chosen intermolecular distance of 2.3 *Å*. The results were obtained with the DFT method and the GW approach for two electric-field polarizations: perpendicular and parallel to the axis of the *π*-stack. The calculated points are marked with circles and the band-lines are interpolated.

The band gap obtained with the DFT method for the molecular wire with the “rotated-shifted” order at  the stacking distance of 2.3 Å is 0.11 eV. This fundamental gap largely opens to 0.4 or 1.16 eV with the GW approach, depending on a polarization of the electric field - parallel or perpendicular to the wire, respectively. Strongly anisotropic GW results for the molecular wires are not surprising^46^. Figure 3 presents the band structures.

**Figure 4:**
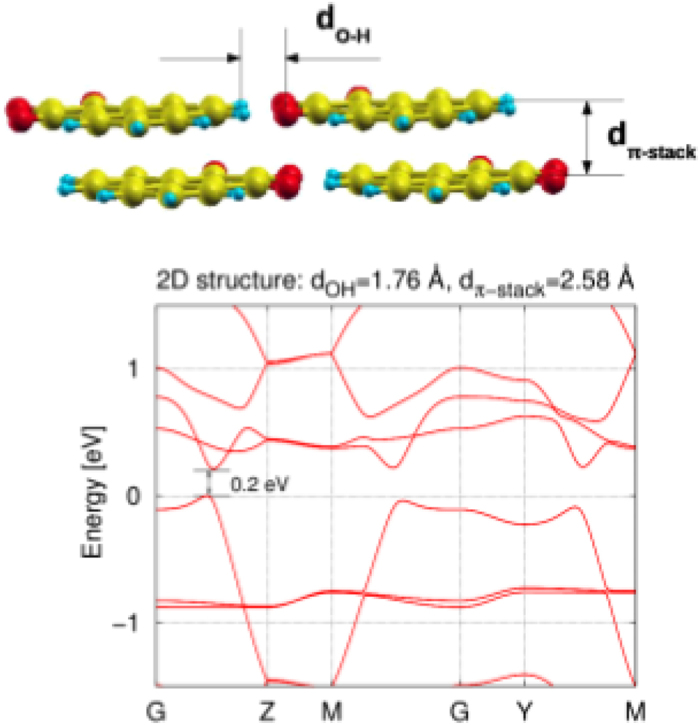
The geometric parameters of the 2D structure formed by a *π*-stacked wires with the “rotated-shifted” molecular order. The planar intermolecular hydrogen-bonds connect the columns. The band structure was obtained with the DFT method for *d*_*π*-*stack*_ = 2.58 *Å* and *d*_*OH*_ = 1.76 *Å*.

The 2D structure is constructed via a repetition of the *π*-stacks along the chosen planar direction - the one which forms the O…H bonds. This is displayed in Fig. S1 in the supporting information (SI). The band structure, plotted for the intermolecular distances of 2.58 and 1.76 *Å* in the stacking and planar O…H-bond directions, respectively, is presented in Fig. 4. While changes of the band structure with a variation of the *d*_*π*-*stack*_ and *d*_*OH*_ parameters are reported in Fig. S2 in SI.

In order to take into account an effect of the exact exchange on the band structure of the 2D case, the hybrid-DFT calculations were performed for the intermolecular distances *d*_*OH*_ = 1.76 and *d*_*π*-*stack*_ = 2.3 *Å*. The fundamental gap obtained with this method is 1.2 eV. A comparison of the hybrid-DFT energy gaps at the symmetry points with the DFT band structure is presented in Fig. S3 in SI.

Most of gaps in TIs open due to the SOC effect. As one would expect, for very light atomic systems, an effect of the spin-orbit coupling is vanishing. The lack of the SOC in the system studied here has been checked with the fully relativistic DFT method. It was necessary, since the low-dimensional systems might be surprising. For example, graphene weakly doped with hydrogen opens the gap to 10^−2^ eV, which is a few orders of magnitude larger than that of pure graphene, 10^−6^ eV^47, 48^. Although the SOC bandgaps in most of the TIs are rather tiny - below 0.1 eV - the values approaching 0.2 eV from the DFT and 0.5 eV from the hybrid-DFT were reported^18, 19^.

**Figure 5:**
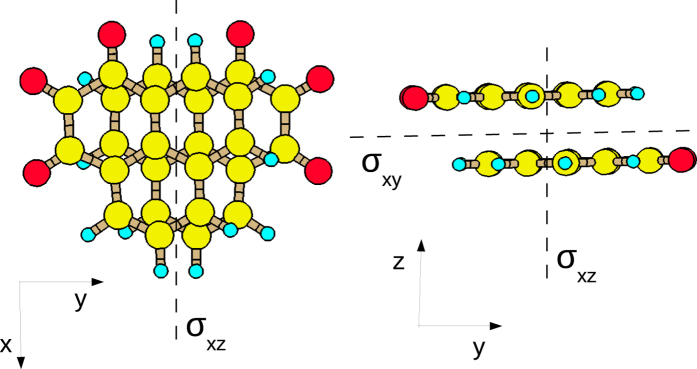
Two mirror planes in the 2D structure formed by the *π*-stacked wires with the “rotated-shifted” molecular order, which are repeated along the hydrogen intermolecular bonds.

The 2D system with “rotated-shifted” molecular order does not possess the rotational symmetry or the inversion in the 3D space. Instead, it transforms with a superposition of two reflections: with respect to (1) the σ_*xy*_ plane, containing the stacking axis and the non-periodic direction, and (2) the σ_*xy*_ plane, which separates the molecules across the *π*-staking axis. The mirror planes are depicted in Fig. 5. This superposition of reflections is equivalent to the inversion in the 2D subspace spanning the periodic directions of the system.

**Figure 6:**
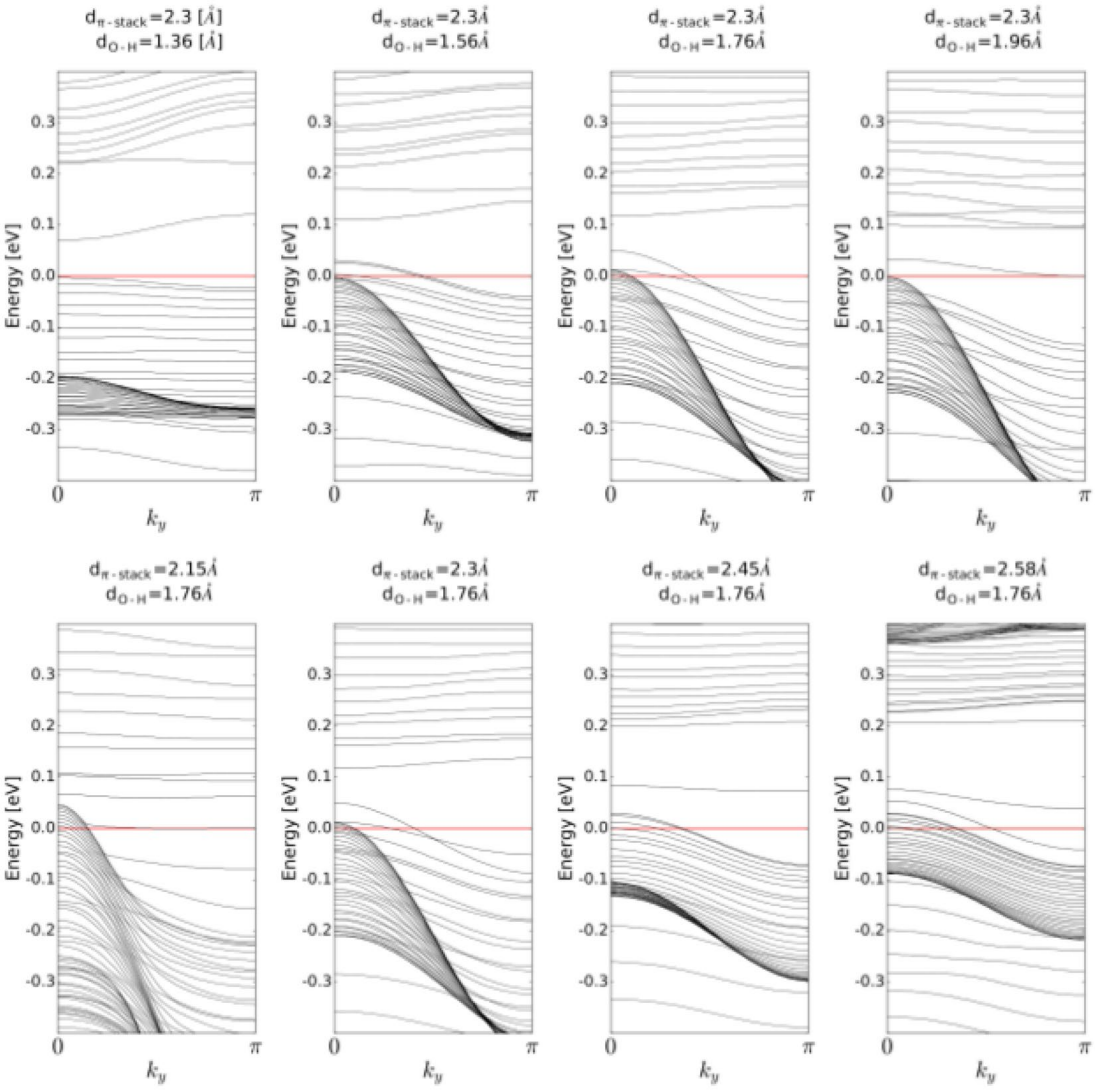
The edge states in the 2D structure presented in Fig. 4. The thickness of the *π*-stacked layers was 50 elementary cells along the z-axis. The y-axis along O-H bonds was treated periodically. The intermolecular distances are given above the corresponding plots.

For the finite system in the stacking direction, the topological crystalline insulating phase is supposed to appear, due to the gapless edge states. They are doubly degenerate with spin components, exhibit the parabolic dispersions, and propagate along the O…H intermolecular bonds. These edge states are tunable with the intermolecular distances, *d*_*π*-*stack*_ and *d*_*OH*_, and are reported in Fig. 6. If the molecular stacking distance is fixed at 2.3 *Å*, and *d*_*OH*_ varies between 1.36 and 1.96 *Å*, the edge states touch the Fermi line from the lower energies at the Γ-point for short O…H, or from the higher energies at the *π*-point for larger distances. While for *d*_*OH*_ in the range [1.56, 1.76] *Å*, the edge states cross the Fermi level. A change of *d*_*π*-*stack*_ to smaller or larger values does not move the edge states away from the Fermi level. In Fig. 6, the finite size along the *π*-stack was set to fifty elementary cells. An evolution of the edge states with a thickness of the stack is presented in Fig. S4 in SI. The pressures corresponding to the studied intermolecular distances are collected in Table 1.

**Table 1:** Pressures (in GPa), obtained with the DFT method for the 2D structure presented in Fig. 4.

*d*_*π*-*stack*_	*P*_*π*-*stack*_	*d*_*OH*_	*P*_*OH*_
2.15	13.7	1.36	2.5
0.30	8.5	1.56	0.3
0.45	5.2	1.60	0.0
0.58	3.3	1.76	−0.9
0.70	1.8	1.96	−1.7

The *d*_*π*-*stack*_ distance (in *Å* was varied when the *d*_*OH*_ distance was fixed to 1.76 *Å*. The dependence on the *d*_*OH*_ distance (in *Å*) was obtained with the fixed *d*_*π*-*stack*_ = 2.3 *Å*.

**Figure 7:**
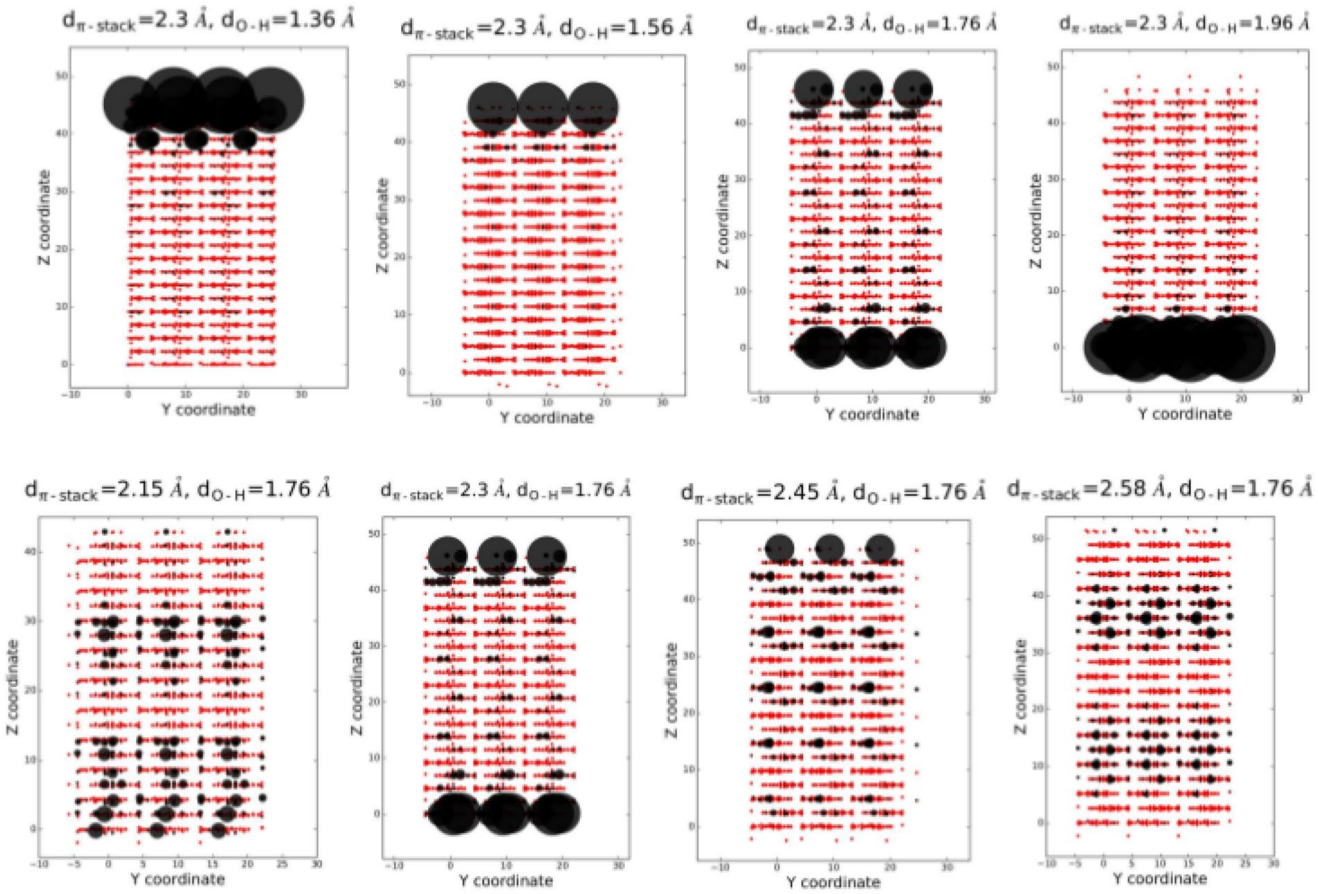
Visualization of the highest-energy occupied eigenvectors of the tight-binding Hamiltonian of the 2D structure presented in Fig. 4. The *π*-stacking and O-H distances were varied. The matrix elements involving the Wannier functions were included when $\langle {W}_{m}|H|{W}_{n}\rangle \ge 0.01\,{\rm{eV}}$.

The localization of the wavefunctions of the edge states can be easily visualized using the Wannier-function^49^ based tight-binding parametrization of the Hamiltonian. In this approach, the periodic problem is solved with constraints for a finite dimension - in this case along the *π*-stack axis. The largest square components of the chosen eigenfunctions are plotted in Fig. 7, for the same *d*_*π*-*stack*_ and *d*_*OH*_ distances which are reported in Fig. 6. There is no exact coincidence between the gapless edge states and the localization at the surface or in the interior. Only one case, *d*_*π*-*stack*_ = 2.3 *Å* and *d*_*OH*_ = 1.76 *Å*, shows the localization of the edge states at the both surfaces of the finite stack. For most of the cases, the edge orbitals localize at one side of the stack. Only for very high- or very low-pressure cases, the edge-state orbitals are delocalized in the interior. For a sake of completeness, the bands, edge states and edge orbitals at ambient pressure (*d*_*π*-*stack*_ = 2.7 *Å* and *d*_*OH*_ = 1.6 *Å*) are presented in Fig. S5 in SI.

Interestingly, the gapless edge-states with the surface-localized orbitals were found for the intermolecular distance *d*_*π*-*stack*_ = 2.3 *Å*. And this stacking distance, taken twice and equal to 4.6 *Å*, was obtained during an optimization of the ferroelectric organic solar cell^50^. The mentioned system was built by benzene rings terminated with the COOH and CH_2_CN dipole groups. These dipoles - of about 0.07 and 0.6 Debye, respectively - tend to orient ferroelectrically in the stacking direction. The COOH group was used to connect molecules in the planar directions, while the CH_2_CN group possessed special transport properties along the stacking axis^50^. The coincidence of the results for the stacking distances suggests, that the CH_2_CN dipole groups could be utilized for a chemical stabilization of the TCI proposed here - if we use them for a connection of every second molecule.

Since the DFT is not a trustworthy method for the orbital order and localization, the pseudopotential self-interaction correction approach^51–53^ (pSIC) has been applied. The pSIC scheme, by its construction, is similar to the LDA + U method. The difference is that all atomic shells (not only *d*- or *f*-type) are corrected, and there is no ‘ad hoc’ parameter such as Hubbard-U. This parameter-free method is an easier alternative to the hybrid-DFT approach, where an amount of the exact exchange can be varied with respect to the applied density-functional exchange. The band structure, edge states and orbitals obtained with the pSIC are presented in Fig. S6 in SI, for the “rotated-shifted” case. Generally, the results qualitatively agree with these from the DFT. However, the pSIC edge states obtained at chosen *d*_*π*-*stack*_ parameters seem to be more correlated with the corresponding DFT results at smaller distances.

**Figure 8:**
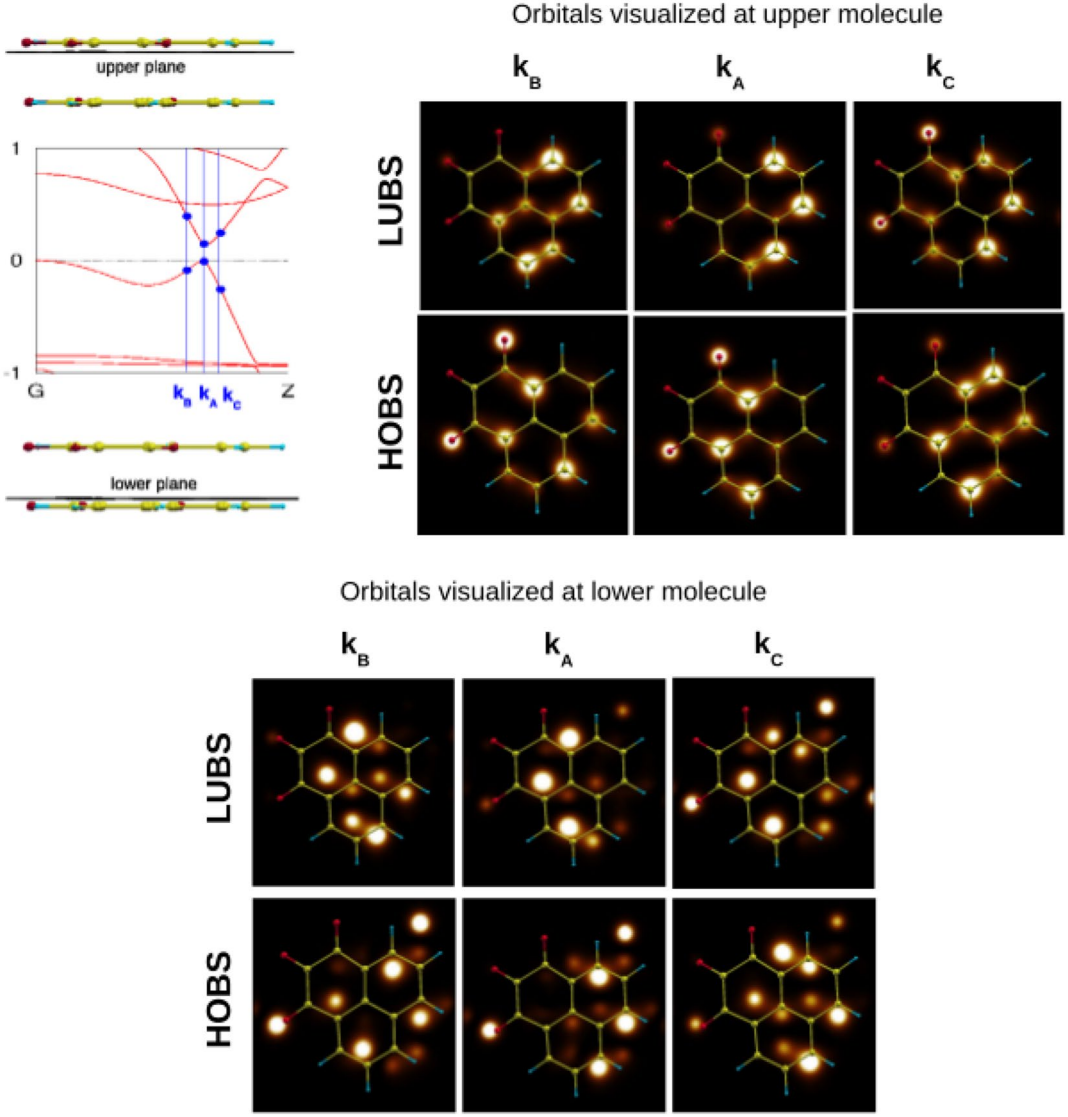
The case with gapless edge states: *d*_*π*-*stack*_ = 2.3 *Å* and *d*_*OH*_ = 1.76 *Å*. Squares of the highest occupied Bloch state (HOBS) and lowest unoccupied Bloch state (LUBS), at three chosen k-points: k_*A*_ = 0.3(3)*π*, k_*B*_ = 0.3 *π*, k_*C*_ = 0.36(6)*π*, are plotted for two chosen 2D slices - just below the upper molecule (M1) and above the lower molecule (M2) in the elementary cell. All plots are done with the same scale for the atomic coefficients. As a reference, a scheleton of the upper molecule is displayed in all panels.

In order to check the bands inversion property, firstly, the elementary cell is divided into “upper” (M1) and “lower” (M2) molecular components. Figure S7 in SI shows the bands projected at the Wannier functions^54^ localized at M1 and M2. The highest occupied Bloch states (HOBS), through whole BZ except the Z-M line, and the lowest unoccupied Bloch states (LUBS) are composed of mixed M1- and M2-centered states in 1:1 ratio. Additionally, in Fig. 8, a composition of HOBS and LUBS with respect to the atomic contributions is mapped at two slices, just below M1 and above M2. This is done for three k-points: at the bandgap (*k*_*A*_) and slightly away (*k*_*B*_ and *k*_*C*_). The scheleton of the M1 molecule is used as a reference (a guide for an eye) for projections at both surfaces. These plots demonstrate only partial bands inversion. The characteristic swap, between HOBS and LUBS for some atomic components, occurs on both sides of the *k*_*A*_ point at the band gap. However, this swapping is more pronounced between *k*_*A*_ and *k*_*C*_ (towards the Z-point) than between *k*_*A*_ and *k*_*B*_ (towards the G-point). Such incomplete, and not shaply marked in BZ, bands inversion is novel among known TIs and TCIs. In almost all topological insulators, the exchange of the bands character takes place between the topological k-points in BZ. However, there is an example in which bands inversion occurs exactly at the topological point and not away from it, namely W_2_HfC_2_O_2_^19^.

Further, an analysis of the symmetry properties of the Bloch functions at the Γ-point and three TRIM (time-reversal invariant momentum) points is done. The manifold of the entangled valence bands is composed of *N*_*val*_ = 50 states. However, at high pressures, the six lower bands merge with the upper fifty states. The energy gap which separates the bottom of the valence band manifold, for all studied cases, is presented in Fig. S8 in SI. The parities ${\xi }_{i}$ of all valence states and the lowest unoccupied state, for all studied cases, are collected in Tables T1 and T2, and T3 in SI. The product of Bloch’s parities, $\delta ={\sum }_{i}^{{N}_{val}}{\xi }_{i}$, is also given. It indicates that the 2D system with “rotated-shifted” order might be a weak topological crystalline insulator at some small pressures. The conclusive data are collected in Table 2.

**Table 2:** Characterization of the valence band parities at the Γ-point and three TRIM points: Z = (0, 0, 1/2), Y = (0, 1/2, 0), M = (0, 1/2, 1/2), for the 2D structures with various [*d*_*π*-*stack*_, *d*_*OH*_] distances (in *Å*).

Case	*N*_*val*_	*δ*(Γ)	*δ*(Z)	*δ*(Y)	*δ*(M)	H/L(Γ)	H/L(Z)	H/L(Y)	H/L(M)	gapless edge st.	localization of edge st.
**DFT**											
2.3, 1.36	50	+	−	+	−	−/−	−/+	−/−	−/+	No	one side
0.3, 1.56	50	+	−	+	−	−/−	−/−	−/−	−/−	Yes	one side
0.3, 1.76	50	+	−	+	−	−/−	−/−	−/−	−/+	Yes	both sides
0.3, 1.96	50	+	−	+	−	−/−	−/+	−/−	−/+	No	one side
2.15, 1.76	56	+	+	+	+	−/−	−/−	−/−	−/−	Yes	delocalized
0.45, 1.76	50	+	−	+	−	−/−	−/+	−/−	−/+	Yes	one side
0.58, 1.76	50	+	−	+	−	−/−	−/+	−/−	−/+	Yes	delocalized
2.70, 1.60	50	+	−	+	−	−/−	+/+	−/−	−/+	Yes	delocalized
**pSIC**											
2.30, 1.76	56	+	+	+	+	−/−	−/+	−/−	+/+	Yes	both sides
0.58, 1.76	50	++	−	+	−	−/−	+/+	−/−	+/+	Yes	one side
0.70, 1.60	50	+	−	+	−	−/−	−/+	−/−	−/+	Yes	delocalized

*N*_*val*_ is a number of the entangled valence bands, *δ* is a product of the Bloch’s parities within the valence-band manifold, H/L means HOBS/LUBS. The rightmost two columns indicate whether the edge states cut the Fermi level or not, and report the localization of these states at the surface or in the interior.

## Conclusion

In this work, a new plausible topological crystalline insulator has been found via the DFT-based tight-binding simulations. This is a 2D molecular system with *π*-stacks in one dimension and hydrogen bonds in the other. It is composed of light elements: C,O,H. The gapless edge states, with the quadratic dispersions, propagate through the hydrogen bonds within the molecular planes, for the finite stacking size. The character of these states - the parity and localization - are tunable with the intermolecular distances *d*_*π*-*stack*_ and *d*_*OH*_. There is completely no effect of the spin-orbit interaction, neither intrinsic nor Rashba type. Only partial bands inversion occurs, and it is not sharply marked in a well defined BZ region. This makes the proposed system to be unique among known or predicted up-to-date TCIs.

## Methods

The density functional theory^55, 56^ calculations were performed employing the Quantum ESPRESSO (QE) suit of codes^57^. It is based on the plane waves and the pseudopotentials describing the atomic cores. The normconserving pseudopotentials were chosen in this work. The energy cutoff of 60 Ry for the plane waves was enough to converge the band structures. The uniform k-mesh of Monkhorst and Pack^58^ with the 12-point grid along the stack and 8-point grid along the OH intermolecular bonds was sufficient for the self-consistent calculations. The exchange-correlation functional in the gradient corrected Perdew-Burke-Ernzerhof parametrization was chosen^59^. The vacuum space between the 2D slabs in the non-periodic direction was set to 30 *Å*.

In order to accurately interpolate the band structures, the wannier90 package^60^ was used. It enables finding the maximally-localized Wannier functions for the composite bands^49, 54^. The Kohn-Sham Hamiltonian^55, 56^ between the Wannier functions^54^, which were obtained from the DFT Bloch functions, was used as a tight-binding model for the edge-states analysis. The cut-off for the nonvanishing matrix elements was set to 0.005 eV. For the tight-binding simulations, the pythTB^61^ code was utilized. The parities were obtained with the bands.x utility from the QE package.

The GW calculations were performed with the Yambo code^62^ in the non self-consistent G_0_W_0_ scheme^63, 64^. The frequency-dependent electronic screening was calculated within the plasmon-pole approximation^65^. A cutoff of 12 Ry was used for the exchange self-energy. The dielectric matrix was constructed summing up to 800 unoccupied bands and using a cutoff of 6 Ry. Up to 1200 unoccupied bands were taken for the G_0_W_0_ summation. For the hybrid-DFT calculations, within the PBE0 flavour^66^, the uniform mesh of the q-vectors with the 3-point grid along the stack and 2-point grid along the OH-bonds was used. This is a commensurate subset of the k-points used in the DFT calculations.

## Electronic supplementary material

Supplementary information

## References

[1] Kane CL, Mele EJ (2005). Z_2_ topological order and the quantum spin hall effect. Phys. Rev. Lett..

[2] Moore, J. E. The birth of topological insulators. *Nature* **464**, 194–198 (210). 10.1038/nature08916 20220837

[3] König M (2007). Quantum spin hall insulator state in hgte quantum wells. Science.

[4] Knez I, Du R-R, Sullivan G (2011). Evidence for helical edge modes in inverted inas/gasb quantum wells. Phys. Rev. Lett..

[5] Fu L, Kane CL (2007). Topological insulators with inversion symmetry. Phys. Rev. B.

[6] Hasan MZ, Kane CL (2010). Colloquium: Topological insulators. Rev. Mod. Phys..

[7] Xiao D (2010). Half-heusler compounds as a new class of three-dimensional topological insulators. Phys. Rev. Lett..

[8] Lin H (2010). Single-dirac-cone topological surface states in the tlbise2 class of topological semiconductors. Phys. Rev. Lett..

[9] Yan B, Zhang SC (2012). Topological materials. Rep. Prog. Phys..

[10] Sun Y (2011). Train-driven onset of nontrivial topological insulating states in zintl sr2 x compounds (x = pb, sn). Phys. Rev. B.

[11] Sun Y, Chen X-Q, Yunoki S, Li D, Li Y (2010). New family of three-dimensional topological insulators with antiperovskite structure. Phys. Rev. Lett..

[12] Lu F, Zhao JZ, Weng H, Fang Z, Dai X (2013). Correlated topological insulators with mixed valence. Phys. Rev. Lett..

[13] Bernevig BA, Hughes TL, Zhang SC (2006). Quantum spin hall effect and topological phase transition in hgte quantum wells. Science.

[14] Murakami S (2006). Quantum spin hall effect and topological phase transition in hgte quantum wells. Phys. Rev. Lett..

[15] Liu Z (2011). Stable nontrivial z2 topology in ultrathin bi (111) films: A first-principles study. Phys. Rev. Lett..

[16] Liu CC, Feng W, Yao Y (2011). Quantum spin hall effect in silicene and two-dimensional germanium. Phys. Rev. Lett..

[17] Qi J, Li X, Qian X (2016). Electrically controlled band gap and topological phase transition in two-dimensional multilayer germanane. Appl. Phys. Lett..

[18] Weng H (2015). Large-gap two-dimensional topological insulator in oxygen functionalized mxene. Phys. Rev. B.

[19] Khazaei M, Ranjbar A, Arai M, Yunoki S (2016). Topological insulators in the ordered double transition metals m’2 m“c2 mxenes (m’ = mo, w; m” = ti, zr, hf. Phys. Rev. B.

[20] Zhao J, Li Y, Ma J (2016). Quantum spin hall insulators in functionalized arsenene (asx, x = f, oh, and ch3) monolayers with pronounced light absorption. Nanoscale.

[21] Ma Y, Kou L, Dai Y, Heine T (2016). Proposed two-dimensional topological insulator in site. Phys. Rev. B.

[22] Liu P-F, Zhou L, Frauenheim T, Wu L-M (2016). New quantum spin hall insulator in two-dimensional mos2 with periodically distributed pores. Nanoscale.

[23] Wang ZF, Liu Z, Liu F (2013). Organic topological insulators in organometallic lattices. Nat. Comm..

[24] Wang A, Zhang X, Zhao M (2014). Topological insulator states in a honeycomb lattice of s-triazines. Nanoscale.

[25] Wang ZF, Su N, Liu F (2013). Prediction of a two-dimensional organic topological insulator. Nano Lett..

[26] Zhou Q, Wang J, Chwee TS, Wu G, Wang X (2015). Topological insulators based on 2d shape- persistent organic ligand complexes. Nanoscale.

[27] Fu L (2011). Topological crystalline insulators. Phys. Rev. Lett..

[28] Chong YD, Wen X-G, Soljacic M (2008). Effective theory of quadratic degeneracies. Phys. Rev. B.

[29] Sun K, Yao H, Fradkin E, Kivelson SA (2009). Topological insulators and nematic phases from spontaneous symmetry breaking in 2d fermi systems with a quadratic band crossing. Phys. Rev. Lett..

[30] Alexandradinata A, Fang C, Gilbert MJ, Bernevig BA (2014). Spin-orbit-free topological insulators without time-reversal symmetry. Phys. Rev. Lett..

[31] Barone P (2013). Pressure-induced topological phase transitions in rocksalt chalcogenides. Phys. Rev. B.

[32] Kargarian M, Fiete GA (2013). Topological crystalline insulators in transition metal oxides. Phys. Rev. Lett..

[33] Sun Y (2013). Rocksalt sns and snse: Native topological crystalline insulators. Phys. Rev. B.

[34] Hsieh TH (2012). Topological crystalline insulators in the snte material class. Nat. Commun..

[35] Tanaka Y (2012). Experimental realization of a topological crystalline insulator in snte. Nat. Phys..

[36] Dziawa P (2012). Topological crystalline insulator states in pn_1−*x*_sn_*x*_se. Nat. Mater..

[37] Wojek BM (2015). Direct observation and temperature control of the surface dirac gap in a topological crystalline insulator. Nat. Commun..

[38] Sessi P (2016). Robust spin-polarized midgap states at step edges of topological crystalline insulators. Science.

[39] Niu C (2015). Two-dimensional topological crystalline insulator and topological phase transition in tlse and tls monolayers. Nano Lett..

[40] Liu J (2014). Spin-filtered edge states with an electrically tunable gap in a two-dimensional topological crystalline insulator. Nat. Mater..

[41] Wrasse EO, Schmidt TM (2014). Prediction of two-dimensional topological crystalline insulator in pbse monolayer. Nano Lett..

[42] Kim Y, Kane CL, Mele EJ, Rappe AM (2015). Layered topological crystalline insulators. Phys. Rev. Lett..

[43] Monserrat, B., Bennett, J. W., Rabe, K. M. & Vanderbilt, D. Antiferroelectric topological insulators in abc compounds. *arXiv:1702.06958* (2017). 10.1103/PhysRevLett.119.036802 28777633

[44] Kindermann M (2015). Topological crystalline insulator phase in graphene multilayers. Phys. Rev. Lett..

[45] http://www.chemspider.com/chemical-structure.72185.html.

[46] Bruneval F, Botti S, Reining L (2005). Comment on “quantum confinement and electronic properties of silicon nanowires”. Phys. Rev. Lett..

[47] Zhou J, Liang Q, Dong J (2010). Enhanced spin–orbit coupling in hydrogenated and fluorinated graphene. Carbon.

[48] Balakrishnan J, Koon GKW, Jaiswal M, Castro Neto AH, Özyilmaz B (2013). Colossal enhancement of spin–orbit coupling in weakly hydrogenated graphene. Nat. Phys..

[49] Marzari N, Mostofi AA, Yates JR, Souza I, Vanderbilt D (2012). Maximally localized wannier functions: Theory and applications. Rev. Mod. Phys..

[50] Wawrzyniak-Adamczewska M, Wierzbowska M (2016). Separate-path electron and hole transport across pi-stacked ferroelectrics for photovoltaic applications. J. Phys. Chem. C.

[51] Filippetti A, Spaldin NA (2003). Self-interaction-corrected pseudopotential scheme for magnetic and strongly-correlated systems. Phys. Rev. B.

[52] Filippetti A, Fiorentini VA (2009). A practical first-principles band-theory approach to the study of correlated materials - self-interaction corrected local-density-functional theory. Eur. Phys. J. B.

[53] Wierzbowska M, Majewski JA (2011). Forces and atomic relaxation in density functional theory with the pseudopotential self-interaction correction. Phys. Rev. B.

[54] Marzari N, Vanderbilt D (1997). Maximally localized generalized wannier functions for composite energy bands. Phys. Rev. B.

[55] Hohenberg P, Kohn W (1964). Inhomogeneous electron gas. Phys. Rev..

[56] Kohn W, Sham LJ (1965). Self-consistent equations including exchange and correlation effects. Phys. Rev..

[57] Giannozzi P (2009). Quantum espresso: a modular and open-source software project for quantum simulations of materials. J. Phys.: Condens. Matter..

[58] Monkhorst HJ, Pack JD (1976). Special points for brillouin-zone integrations. Phys. Rev. B.

[59] Perdew JP, Burke K, Ernzerhof M (1996). Generalized gradient approximation made simple. Phys. Rev. Lett..

[60] Mostofi AA (2008). wannier90: A tool for obtaining maximally-localised wannier functions. Comput. Phys. Commun..

[61] Coh, S. & Vanderbilt, D. Python tight binding (pythtb) code, available at http://www.physics.rutgers.edu/pythtb/index.html.

[62] Marini A, Hogan C, Grüning M, Varsano D (2009). Yambo: an ab initio tool for excited state calculations. Comp. Phys. Comm..

[63] Aryasetiawan F, Gunnarsson O (1998). The gw method. Rep. Prog. Phys..

[64] Schindlmayr A, Garcia-Gonzalez P, Godby RW (2001). Diagrammatic self-energy approximations and the total particle number. Phys. Rev. B.

[65] Godby RW, Needs RJ (1989). Metal-insulator transition in kohn-sham theory and quasiparticle theory. Phys. Rev. Lett..

[66] Perdew JP, Ernzerhof M, Burke K (2007). Rationale for mixing exact exchange with density functional approximations. J. Chem. Phys..

