# Supplementary material for: Gapless edge states in (C,O,H)-built molecular system with p-stacking and hydrogen bonds
Source: Sci Rep. 2017 Aug 29;7:9888. doi: 10.1038/s41598-017-09954-z (PMC5575060; doi:10.1038/s41598-017-09954-z)
Supplement: Supplementary file 1 — Supplementary information [file 41598_2017_9954_MOESM1_ESM.pdf]

SUPPORTING INFORMATION

Gapless edge states in the (C,O,H)-built molecular system with π-stacking and hydrogen bonds

Małgorzata Wierzbowska

Institute of High Pressure Physics, Polish Academy of Sciences, Sokołowska 29/37, 01-142 Warsaw, Poland

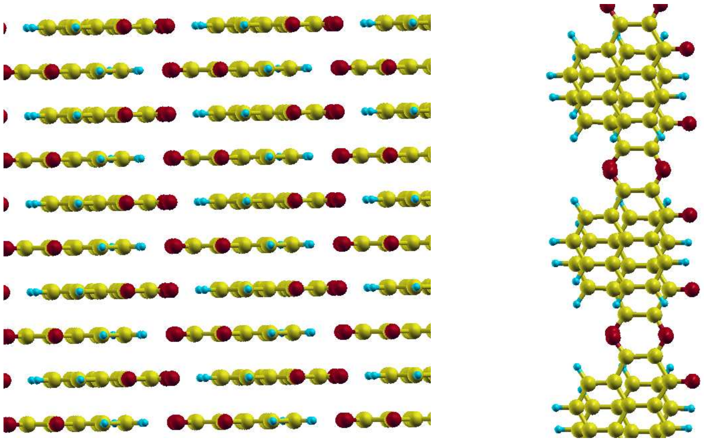

FIG. 1: The geometry of the 2D structure called "rotated-shifted", formed by columns of the pi-stacked molecules. The columns are repeated periodically in the direction where the hydrogen bonds form between the molecules.

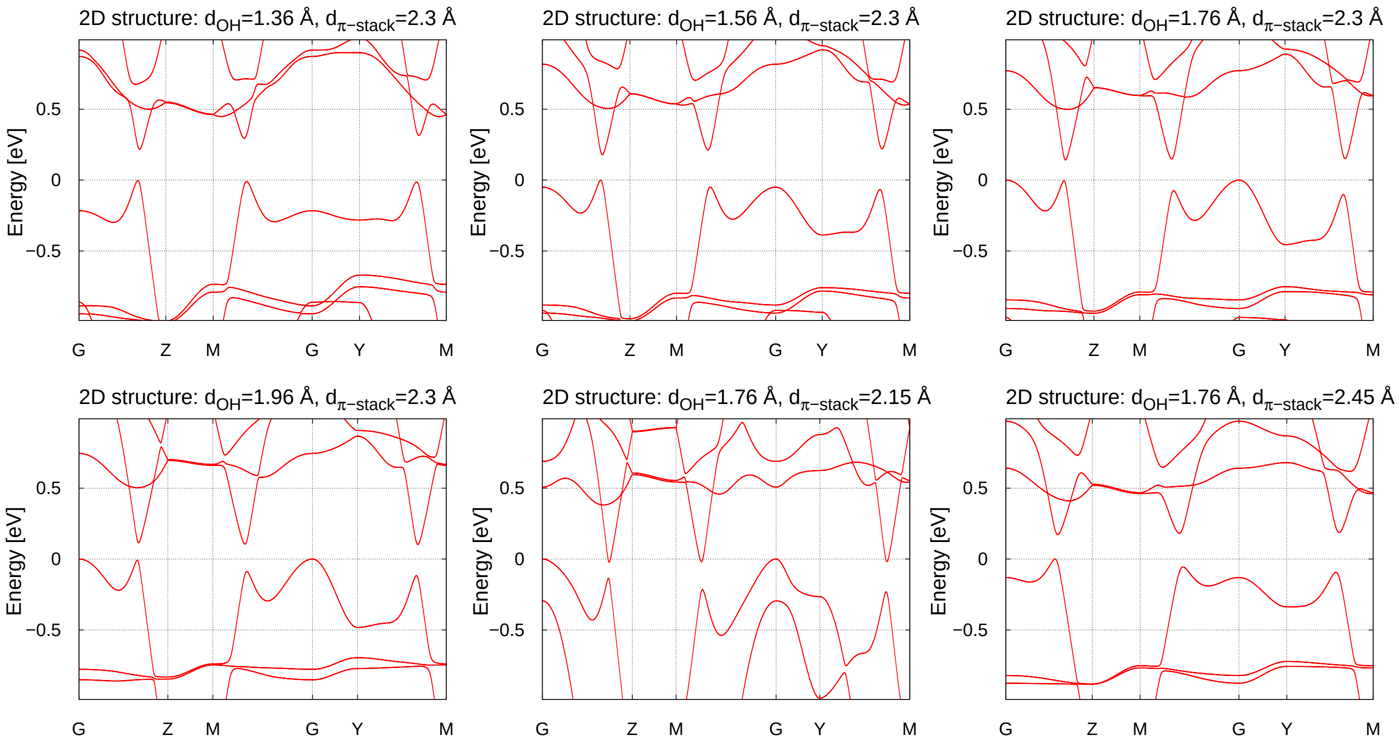

FIG. 2: The band structures of the 2D structure called "rotated-shifted". The parameters d_{OH} and $d_{\pi-stack}$ are varied and given above each plot.

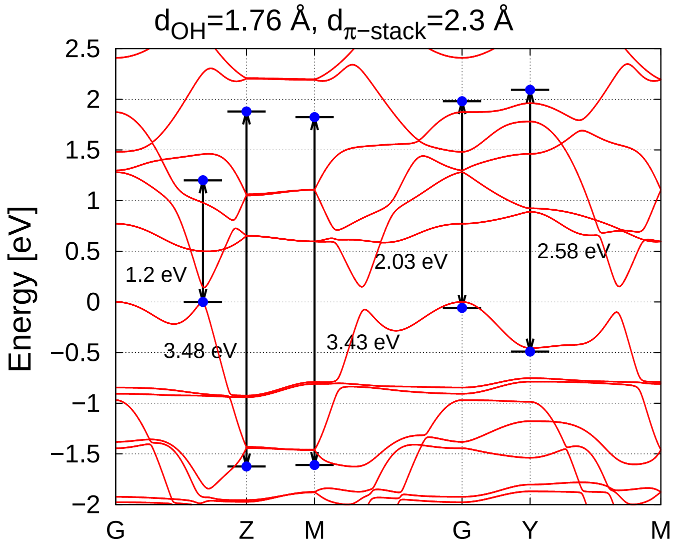

FIG. 3: The fundamental gap and the energy gaps at the symmetry points obtained with the hybrid-DFT (blue dots) in a comparison with the DFT band lines.

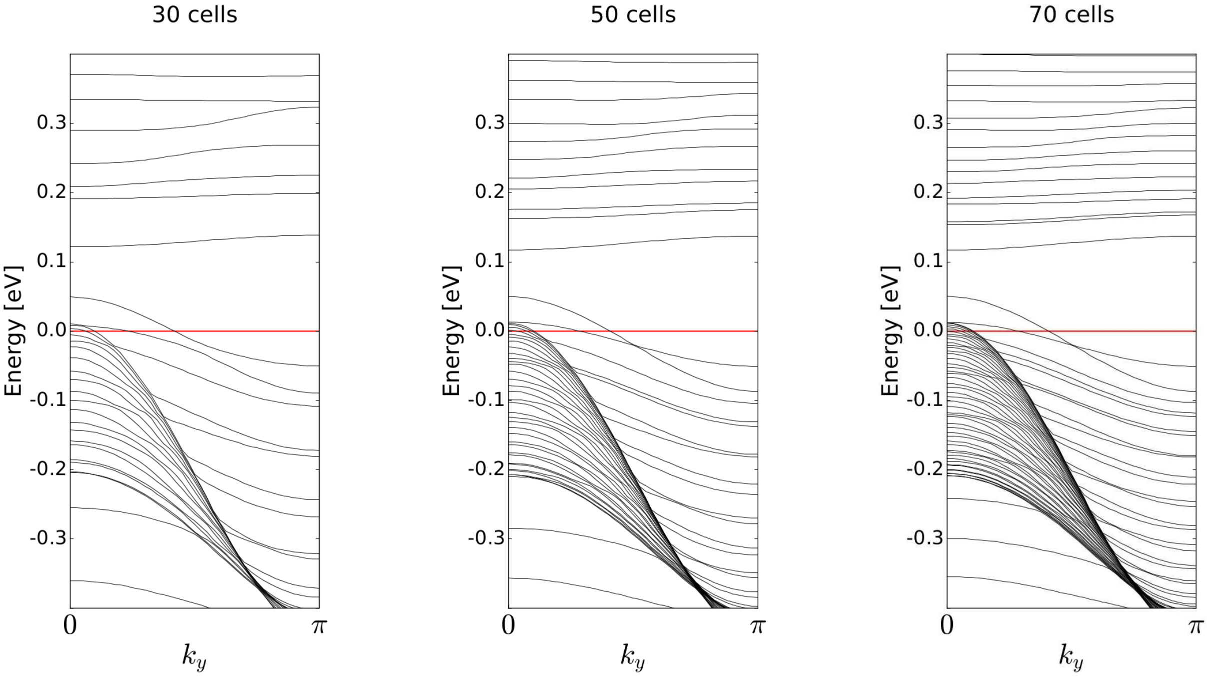

FIG. 4: The edge states obtained with the DFT method for the 2D structure called "rotated-shifted", with the intermolecular parameters $d_{\pi-stack}$=2.3 $\AA$ and d_{OH}=1.76 $\AA$. The number of elementary cells in the stacking direction was varied: 30, 50, 70. The direction along the hydrogen bonds was treated periodically.

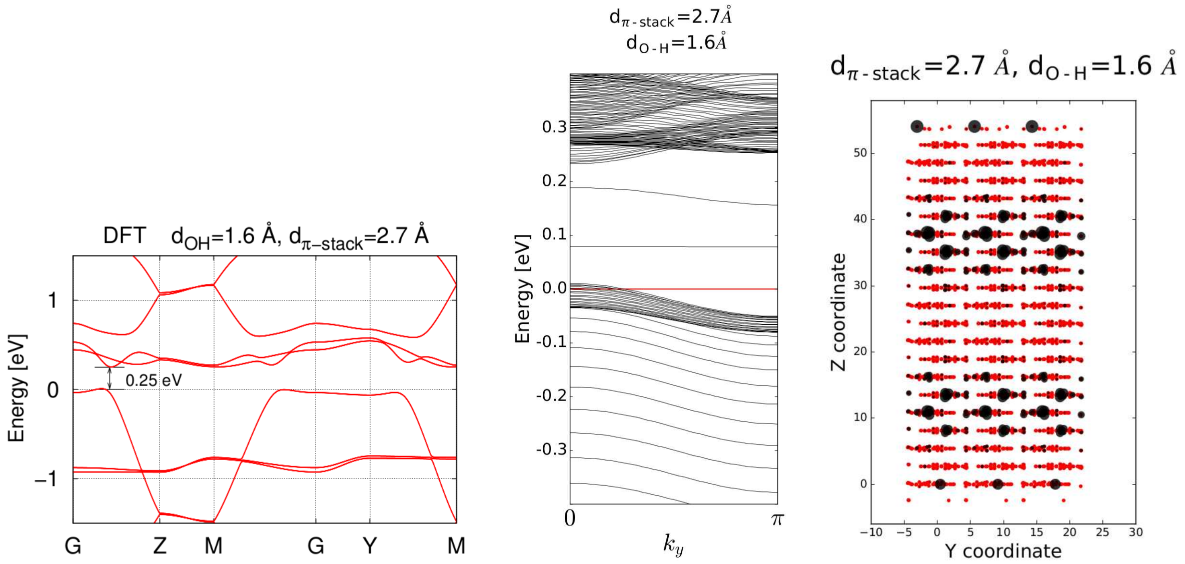

FIG. 5: the band structure, edge states and visualization of the highest occupied orbital. The results were obtained with the DFT method for the 2D structure called "rotated-shifted".

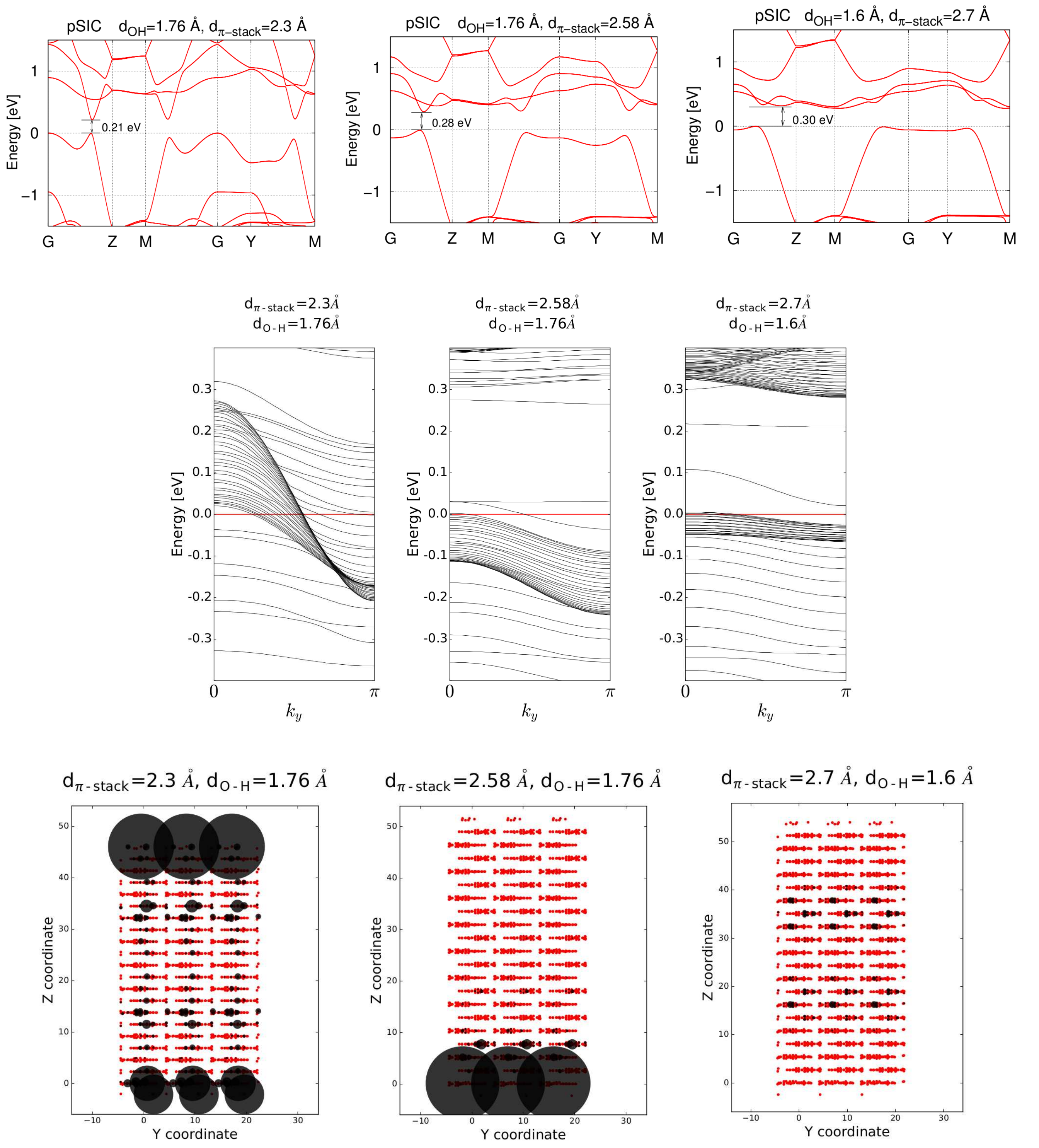

FIG. 6: The results obtained with the pSIC method: band structure, edge states and visualization of the highest occupied orbital. The intermolecular distances are given above the corresponding panels.

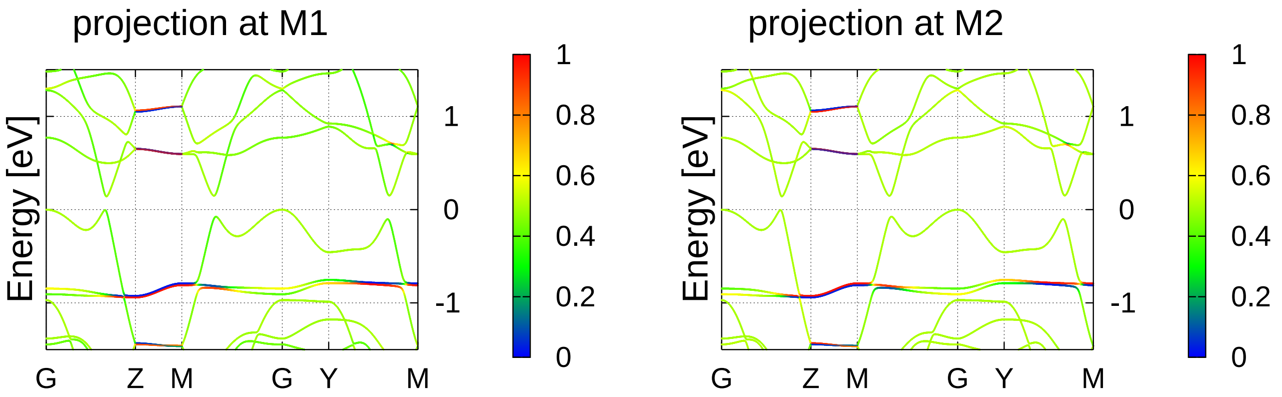

FIG. 7: The band lines of the 2D structure called "rotated-shifted". Colour scale shows the coefficient of a projection onto the Wannier functions which are localized at the upper/lower molecule in the cell (named M1/M2, respectively). Results were obtained with the DFT method for the intermolecular π-stacking distance 2.3 $\AA$ and O-H bond 1.76 $\AA$.

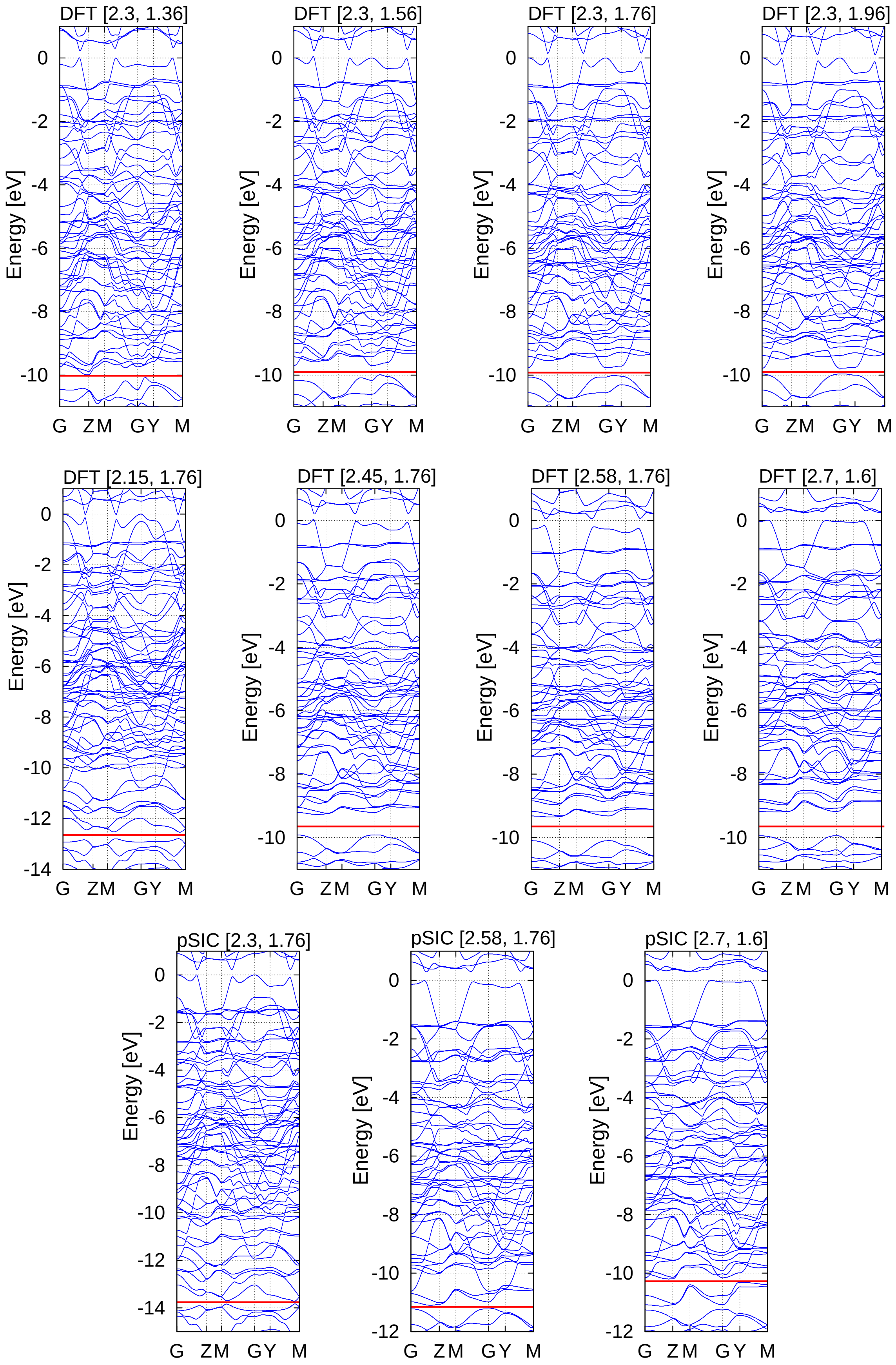

FIG. 8: The manifold of the entangled valence bands for all reported cases of the intermolecular distances in the 2D structure called "rotated-shifted". The bottom of the valence states is marked with the red line. The Fermi level is at zero energy. The method of the calculation and the intermolecular distances [$d_{\pi-stack}$,d_{OH}] are given above the corresponding panels.

TABLE I: Parities of the Bloch functions, for a few values of the d_{OH} parameter and fixed $d_{\pi-stack}$=2.3Å, obtained at the Γ point and three TRIM points: Z=(0,0,1/2), Y=(0,1/2,0) and M=(0,1/2,1/2). The parameter δ is a product of parities for all valence states. Results were obtained with the DFT method.

Case	K-point	valence states ; LUBS	δ
d_{OH}=1.36Å	Γ	- + + + + + + + - + + - - + + - + + + + + + + - +	
$d_{\pi-stack}$=2.3Å		- + + + + + + - + - + + - - - + + + + - - + + - - ; -	+
	Z	- + + - - + - + - + - + + - + - - + + - + - + - -	
		+ + - + - - + - + - + - + + - + - - + + - - + + - ; +	-
	Y	- + + + + - + + + + - + + - + + + + + - + + + - +	
		+ - + - + + + + + + + - - - + - + + + - - - + + - ; -	+
	M	- + - + + - + - - + - + - + + - + - - + + - + - -	
		+ + - + - + - - + - + - + + - + - + - - + + - + - ; +	-
d_{OH}=1.56Å	Γ	- + + + + + + + - + + - - + + + - + + + + + + - +	
$d_{\pi-stack}$=2.3Å		+ - + + + + + + - + - + - - - + + + + - - + - + - ; -	+
	Z	+ - - + - + + - - + - + + - + - + - - + + - + - +	
		- - + + - - + - + - + - + + - + - - + + - - + + - ; -	-
	Y	- + + + + + - + + + - + + + + - + + + - + + + - +	
		+ - + - + + + + + + + - - - + + - + + - - - + + - ; -	+
	M	- + + - + - - + - + - + - + + - + - - + + - + - -	
		+ + - + - - + - + - + - + + - + - - + - + + - + - ; -	-
d_{OH}=1.76Å	Γ	- + + + + + + + - + + - - + + + + + - + + + + + -	
$d_{\pi-stack}$=2.3Å		+ - + + + + + + - + - + - - - + + + + - - - + + - ; -	+
	Z	+ - - + - + + - - + - + + - + - + - + - - + + - +	
		- - + + - - + - + - + - + + - + - - + + - - + + - ; -	-
	Y	- + + + + + + - + + - + + + + - + + + + + - + - +	
		- + + + - + + + + + + - - - + + - + + - - - + + - ; -	+
	M	- + + - + - - + - + + - - + + - + - - + + + - - +	
		- - + + - - + - + - + - + + - + - - + - + + - + - ; +	-
d_{OH}=1.96Å	Γ	- + + + + + + + - + - + + - + + + + + + - + + - +	
$d_{\pi-stack}$=2.3Å		+ - + + + + + - + + + - - - - + + + + - - - + + - ; -	+
	Z	+ - - + - + + - - + + - + - + - + - + - + - + - +	
		- - + - + - + + - - + - + + - + - - + + - - + + - ; +	-
	Y	- + + + + + + - + - + + + + + + + - + + + + - - +	
		- + + + + - + + + + + - - - + + - + + - - - + + - ; -	+
	M	- + + - + - + - - + + - - + + - + - - + + - + - +	
		- - + + - - + - + - + - + + - + - - + + - + - + - ; +	-

TABLE II: Parities of the Bloch functions, for a few values of the $d_{\pi-stack}$ parameter, at the Γ point and three TRIM points: Z=(0,0,1/2), Y=(0,1/2,0) and M=(0,1/2,1/2). The parameter δ is a product of parities for all valence states. The case with $d_{\pi-stack}$=2.15Å has a larger manifold of entangled valence states. Results were obtained with the DFT method.

Case	K-point	valence states ; LUBS	δ
d_{OH}=1.76Å	Γ	+ + + + + -	
		+ + + + - + + + + - + + - + - + + + + + + - + - +	
$d_{\pi-stack}$=2.15Å		+ + + + + + + + - + - + - - + - + + + - - + + - - ; -	+
	Z	+ - + - - +	
		+ - - + - + + - - + - + + - + - + - + - + - + - +	
		- - + - + + - - + - + - + + - + - - + + - - + + - ; -	+
	Y	+ + + + - +	
		+ + + + + - - + + + + + + + - + - + + + - + + + -	
		+ - + + + + + + + + + - - - + + - + - + - + + - - ; -	+
	M	+ - - + - +	
		- + + - + - + - + - + - - + + - + - - + + - + + -	
		- - + - + - + - + - + - + + - + - - + - + + - + - ; -	+
d_{OH}=1.76Å	Γ	+ - + + + + + + + - + - + - + + + + + + + - + + +	
$d_{\pi-stack}$=2.45Å		- + + - + + + - + + + - - - + - + + + - - - + + - ; -	+
	Z	+ - - + - + + - - + - + + - + - + - - + + - - + -	
		+ + - - + - + - + + - - + + - + - - + + - - + + - ; +	-
	Y	+ - + + + + + + - + + + - + + + + + - + + + - + +	
		- - + + + + - + + + + - - - + + - + - + - - + + - ; -	+
	M	- + + - + - - + - + + - - + + - + - - + + - + - -	
		+ + - + - + - - + - + - + - + + - - + + - + - + - ; +	-
d_{OH}=1.76Å	Γ	- + + + + + + + - + - + + - + + + + + + - + + - +	
$d_{\pi-stack}$=2.58Å		+ - + + + + + - + + + - - - - + + + + - - - + + - ; -	+
	Z	+ - - + - + + - - + + - + - + - + - + - + - + - +	
		- - + - + - + + - - + - + + - + - - + + - - + + - ; +	-
	Y	- + + + + + + - + - + + + + + + + - + + + + - - +	
		- + + + + - + + + + + - - - + + - + + - - - + + - ; -	+
	M	- + + - + - + - - + + - - + + - + - - + + - + - +	
		- - + + - - + - + - + - + + - + - - + + - + - + - ; +	-
d_{OH}=1.6Å	Γ	+ + + + + - + + + + - + + + - + - + + + + + - + +	
$d_{\pi-stack}$=2.7Å		+ - + + + - - + + + - + - - + + - - + + - - + + - ; -	+
	Z	+ - - + - + - + - + - + - + - + + + - - + - - + -	
		+ + - + - - + + - - + + - + - + - - + + - - + - + ; +	-
	Y	+ + + + + - + + + - + + + + + + - + + - + + + + -	
		- + + + - + - + + + - + - - + - + - + - + - + + - ; -	+
	M	- + + - - + + - - + - + - + - + + - + - - + + - -	
		+ - + + - - + + - - - + + - + + - - + + - + - + - ; +	-

TABLE III: Parities of the Bloch functions, at the Γ point and three TRIM points: Z=(0,0,1/2), Y=(0,1/2,0) and M=(0,1/2,1/2). The parameter δ is a product of parities for all valence states. The case with $d_{\pi-stack}$=2.3Å has a larger manifold of entangled valence states. Results were obtained with the pSIC method.

Case	K-point	valence states ; LUBS	δ
d_{OH}=1.76Å	Γ	+ + + + - +	
$d_{\pi-stack}$=2.3Å		+ + + + + + - + + - - + + + - + + + + + + + - + +	
		- + + + + + + - + + - + - + + - - + + + - + - - - ; -	+
	Z	- + + - - +	
		+ - - + - + + - - + - + + - + - + - + - - + + - +	
		- - + + - - + - + + - - + + - + - - + - + - + + - ; +	+
	Y	+ + + + - +	
		+ + + + + - + - + + + + + + - + + - + + + + + - +	
		- + - + + + + + + + + - - - + + + + - - + + - - - ; -	+
	M	+ - - + + -	
		- + + - - + + - - + + - - + + - + - - + + - + - +	
		- - + + - - + - + + - - + + - + - - + - + + - - + ; +	+
d_{OH}=1.76Å	Γ	+ + - + + + + + + - + - - + + + + + + - + + + + +	
$d_{\pi-stack}$=2.58Å		- + + - + + - + + + + - - + + - + + - - - + - + - ; -	+
	Z	- + - + - + + - - + + - - + - + - + - + - + - + +	
		- + - + - - + + - - + + - + - + - + - - + - + - + ; +	-
	Y	+ + + + + + + + + + + - + + + + + - + + + + - + +	
		- - + + + - + + + + + - - - + + + + - - - - + + - ; -	+
	M	- + + - + - - + + - - + + - - + + - - + - + - + -	
		+ + - + - - + - + - + - + + - + - - + - + + - - + ; +	-
d_{OH}=1.6Å	Γ	+ + - + + + + + + - + + - - + + + + - + + + + + +	
$d_{\pi-stack}$=2.7Å		- + + + + - - + + - + + - + - + + + - - - - + + - ; -	+
	Z	+ - - + - + - + - + - + - + - + + - + - + - + - -	
		+ + - - + - + - + - + - + + - + - - + - + - + - + ; -	-
	Y	+ + - + + + + + - + + + + + - + + - + + + + - + +	
		- + - + + - + + + - + + - - + + - + - + - - + + - ; -	+
	M	- + + - - + + - - + - + + - - + + - + - - + + - +	
		- - + - + - + + - - + - + + - + - - + + - + - + - ; +	-
